# Digital government and residents’ mental health in China: evidence on potential mechanisms and urban–rural heterogeneity

**DOI:** 10.3389/fpubh.2026.1872844

**Published:** 2026-06-26

**Authors:** Hao Ji, Xianguo Qu, Weiguang Pan, Junyi Xin, Yingying Yu

**Affiliations:** 1 Medical Information Center, Hangzhou Medical College, Hangzhou, China; 2 School of Economics and Management, Zhejiang A&F University, Hangzhou, China; 3 School of Public Health, Zhejiang University, Hangzhou, China; 4 Zhejiang Institute of Rural Revitalization, Hangzhou, China; 5 School of Information Engineering, Hangzhou Medical College, Hangzhou, China

**Keywords:** digital government, health-related activity limitation, life happiness, mental health, urban–rural heterogeneity

## Abstract

**Background:**

Digital government has become a critical component of contemporary public governance, yet its relationship with residents’ mental health remains insufficiently examined. This study investigates whether digital government development is associated with residents’ mental health in China and how this association varies across urban and rural populations.

**Methods:**

We linked the 2020 and 2022 provincial Digital Government Development Indices with individual-level data from the 2021 and 2023 waves of the Chinese General Social Survey. Employing a one-period lagged matching strategy within a pooled cross-sectional design, we estimated the association using Ordinary Least Squares (OLS) models. Instrumental variable (IV) estimation was used to address potential endogeneity concerns, supplemented by robustness checks, mechanism analysis, and heterogeneity analysis.

**Results:**

Digital government development is positively and consistently associated with residents’ mental health. Mechanism analyses suggest that reduced health-related activity limitations and enhanced life happiness may serve as two potential transmission pathways. This positive association appears more stable among rural residents, although the statistical significance of the urban–rural difference should be interpreted cautiously. Further heterogeneity analysis indicates that the association is more evident among individuals with higher human capital, fewer health constraints, and stronger capacity to use digital public services.

**Conclusion:**

Digital government may have implications beyond administrative efficiency by being linked to residents’ mental health, health capabilities, and psychological resources. To foster inclusive digital governance, policymakers should prioritize health service integration, digital accessibility, service usability, and offline support for vulnerable and structurally disadvantaged groups.

## Introduction

Digital government has become an important institutional context for the transformation of contemporary public governance. Data science, artificial intelligence, and the digitalization of public administration are reshaping government organization, governance capacity, and public service delivery in systematic ways (1–3). Digital government is not merely a technological overlay on the public sector. Rather, it refers to a broader transformation in which governments rely on online service platforms, integrated data systems, and digital channels of interaction to restructure public service provision, administrative coordination, and state–citizen interactions (4, 5). As digital technologies become increasingly embedded in public governance, the social implications of digital government have moved beyond technical applications and administrative convenience, becoming increasingly intertwined with citizens’ daily lives, institutional experiences, and individual wellbeing (6, 7). Understanding the relationship between macro-level digital governance transformation and micro-level resident wellbeing has therefore become an important issue across public administration, social policy, and public health.

In the analytical framework of this study, digital government is conceptualized as a macro-level governance environment that may reshape residents’ everyday institutional experiences. In traditional bureaucratic systems and public service settings, residents often face information asymmetry, fragmented procedures, and high offline transaction costs. These institutional frictions not only increase the time costs and cognitive burden associated with handling public affairs but also constitute a form of hidden administrative burden. Through repeated state–citizen interactions, such burdens may be transformed into frustration, helplessness, and persistent psychological stress (8, 9). By contrast, digital government may reduce institutional barriers to accessing public services through data sharing and process reengineering, thereby enabling citizens to obtain more transparent, convenient, and predictable service experiences (4, 10). In this sense, digital government not only changes the form of public service delivery but may also be linked to individual mental health by reducing friction in everyday institutional experiences.

Although existing literature has provided an important foundation for examining the relationship between digital governance and individual wellbeing, several theoretical gaps remain. Previous studies have widely documented positive associations between e-government maturity, digital governance capacity, subjective wellbeing, and life satisfaction (6, 11). However, these studies have mainly focused on general cognitive evaluations, such as service satisfaction and quality of life, while paying insufficient attention to mental health, an individual state with greater public health significance and stronger sensitivity to external environments. Compared with general life satisfaction, mental health more directly reflects emotional stress, depressive feelings, and psychological vulnerability when individuals face uncertainty. Focusing on mental health, therefore, helps provide a more nuanced understanding of the social connection between digital government as a macro-level governance environment and individuals’ micro-level psychological states.

Furthermore, the mechanisms through which a macro-level digital governance environment is linked to individual mental health remain insufficiently theorized. Drawing on health behavior research and positive psychology, this study proposes that digital government may be associated with mental health through two main pathways: the alleviation of objective physiological constraints and the accumulation of subjective psychological resources. First, by improving the accessibility of health and public services, digital government may reduce residents’ health-related activity limitations. Online consultations, electronic health records, and digital tracking systems can lower the information and transaction costs of medical services, making preventive interventions and continuous care more accessible (12, 13). The alleviation of physical functional constraints may help maintain individuals’ independence and sense of control, thereby reducing psychological distress associated with physical limitations. Second, by improving government service experiences and facilitating channels for participation, digital government may enhance residents’ life happiness. Convenient institutional responses may improve individuals’ positive evaluations of their living conditions (14). A higher level of life happiness, as an important psychological resource, may strengthen emotional regulation and psychological resilience and may further be associated with better mental health.

More importantly, the relationship between digital government and mental health may not be evenly distributed across different social spaces. In China, the urban–rural dual structure shaped by the household registration system, or Hukou, has profoundly structured the spatial allocation of public services. Traditionally, agricultural Hukou holders have faced higher spatial barriers and institutional frictions in accessing medical care, social security, and other public resources. Drawing on Amartya Sen’s capability approach (15), this study argues that digital government may have a certain degree of compensatory empowerment significance in narrowing such gaps. By reducing physical and temporal constraints, digital government may create greater marginal room for improvement among agricultural Hukou residents who were previously constrained by weaker public service accessibility. Although the digital divide may weaken these potential gains (16), differences in the baseline conditions of public service provision suggest that the positive association between digital governance and mental health may be more evident or more stable among agricultural Hukou groups. This heterogeneity, however, requires empirical examination.

China’s ongoing digital transformation provides an important empirical setting for investigating these issues. In recent years, China has steadily promoted digital government construction, with online government services, data-sharing platforms, and smart governance tools becoming increasingly widespread. At the same time, across China’s vast geographical space and complex Hukou structure, residents continue to display substantial heterogeneity in resource endowments, public service accessibility, and digital capabilities. The coexistence of rapid digital governance development and structural social disparities makes China a valuable case for examining the relationship between digital government and residents’ mental health, the potential mechanisms underlying this relationship, and its group-based differences.

Against this background, this study links the provincial Digital Government Development Index with micro-level data from the Chinese General Social Survey (CGSS) to address three core questions. First, is digital government development positively associated with residents’ mental health? Second, do health-related activity limitation, as an objective constraint, and life happiness, as a subjective psychological resource, constitute potential transmission mechanisms between digital government and mental health? Third, does this association exhibit a compensatory empowerment pattern, in the sense that the positive association may be more stable or more evident among residents with agricultural Hukou?

This study makes three main contributions. First, by crossing the disciplinary boundaries between public administration and public health, it extends research on digital government and resident wellbeing from administrative efficiency, service convenience, and life satisfaction to the more specific domain of mental health. Second, by incorporating both objective behavioral constraints and subjective cognitive evaluations, it attempts to open the black box linking macro-level digital governance environments to micro-level psychological states. Third, by introducing the theoretical perspective of compensatory empowerment, this study examines heterogeneity in the association between digital government and mental health across Hukou groups, thereby providing new empirical evidence for understanding the relationships among public service equalization, inclusive governance, and residents’ mental health in the digital era.

## Theoretical framework and research hypotheses

### Digital government development and residents’ mental health: the main association hypothesis

Digital government development is not merely the simple addition of information and communication technologies to the public sector. Rather, it is a process through which governments rely on online services, data coordination, and state–citizen interaction platforms to transform modes of public governance and public service delivery (4–6). Along with this process, the focus of digital government research has gradually shifted from early concerns with technology adoption, administrative efficiency, and online service provision to broader issues of citizen experience, subjective wellbeing, and public value creation (6, 7, 17). From a public value perspective, the significance of digital government lies not only in improving the operational efficiency of government but also in enhancing residents’ institutional experiences in state–citizen interactions by improving the accessibility, transparency, and responsiveness of public services.

Such improvements in institutional experience provide an important basis for understanding the relationship between digital government and residents’ mental health. Residents’ mental health is not an isolated individual psychological phenomenon; rather, it is embedded in their everyday living environment and broader public governance context. Under traditional bureaucratic service models, information asymmetry, fragmented procedures, and high offline transaction costs tend to generate institutional frictions in residents’ access to public services (9). These frictions not only increase the time costs and cognitive burden involved in handling public affairs but may also intensify feelings of uncertainty and gradually become chronic stressors in everyday life (8). By contrast, digital government may reduce such institutional frictions through online service platforms, cross-departmental data sharing, process integration, and information disclosure, making access to public services more timely, transparent, and predictable (4, 10). As a result, residents’ frustration, helplessness, and uncertainty when dealing with public affairs may be alleviated, while their sense of control and psychological security may be strengthened.

Existing empirical research also provides preliminary evidence for a positive relationship between digital government and resident wellbeing. Based on panel data from ASEAN countries, Boonratmaitree et al. (11) find that e-government adoption is significantly associated with national happiness. Fan et al. (4), focusing on e-government efficiency, reveal an association between digital government service experience and subjective wellbeing. Khan (6, 18), using cross-national data, further shows that e-government maturity is positively related to national-level wellbeing. Although these studies mainly focus on broader subjective wellbeing, life satisfaction, or overall quality of life rather than directly examining mental health, they collectively suggest that digital government, by improving public service experiences and the governance environment, may be linked to individual mental health gains. Based on these arguments, this study proposes the following hypothesis:

*H1*: Digital government development is positively associated with residents’ mental health.

### Cross-domain physiological–psychological protection: health-related activity limitation as a potential transmission mechanism

Individual physiological functioning constitutes an important foundation of mental health. By reshaping health service delivery networks and facilitating a shift in public health governance from passive ex post responses toward proactive and precise prevention, digital government may contribute to mental health through the reduction of residents’ health-related activity limitations. In this sense, digital government may generate a form of cross-domain protection that links physiological functioning to psychological wellbeing.

On the one hand, the “electronic bridge” constructed by digital government (12) may lower the threshold for accessing health resources. Under traditional health service delivery models, information barriers, transaction costs, identification costs, and application costs often constrain residents’ effective use of public health services (19–21). Through digital appointment systems, telemedicine, health information dissemination, and the interoperability of electronic health records, digital government can help break down information silos in traditional medical systems (22, 23) and reduce the costs associated with accessing preventive health services and continuous care. At the same time, data-driven health monitoring, risk identification, and earlier intervention may enable key groups, such as patients with chronic diseases and older adults, to receive continuous management before health conditions deteriorate (24, 25). Accordingly, digital government may help slow the deterioration of residents’ physiological functioning and reduce the risk of limitations in both basic activities of daily living and instrumental activities of daily living (26).

On the other hand, the alleviation of health-related activity limitations may help interrupt the translation of physiological impairment into psychological distress. From a medical sociology perspective, physical activity limitation is not merely a form of functional decline; it can also constitute a “biographical disruption” in the individual life course (27) and a chronic stressor that contributes to psychological distress, including depressive symptoms (28). Health-related activity limitations may weaken individuals’ independence and self-efficacy, increase social isolation, emotional dependence, and potential stigmatizing experiences (29–31), and thereby reinforce a negative pathway from physiological constraint to social withdrawal and psychological distress. By improving the accessibility, continuity, and precision of health services, digital government may reduce residents’ health-related activity limitations, help maintain their social independence and sense of control over daily life, and thereby mitigate mental health risks associated with physical functional constraints. Based on these arguments, this study proposes the following hypothesis:

*H2*: Health-related activity limitation may constitute a potential transmission mechanism between digital government development and residents’ mental health. Specifically, digital government development is expected to be associated with lower levels of health-related activity limitation, and the alleviation of such limitations is further expected to be associated with better mental health.

### Cognitive–emotional resource transformation: life happiness as a potential transmission mechanism

Within a people-centered framework of modern governance, digital government development has increasingly shifted from an efficiency-oriented agenda toward a public value and citizen wellbeing orientation (17, 32). Life happiness, commonly reflected in life satisfaction, refers to individuals’ long-term cognitive evaluation of their overall quality of life (33, 34). More specifically, digital government may transform positive governance experiences into residents’ life happiness through three main dimensions.

First, digital government may improve service experiences. Through process integration, online service provision, and cross-departmental coordination, digital government can reduce the spatial, temporal, and informational costs associated with accessing public services, thereby enhancing residents’ perceived convenience (5, 6). Second, digital government may improve governance evaluations. Information disclosure, digitized administrative procedures, and online oversight mechanisms can help strengthen administrative transparency and accountability, thereby increasing public recognition of governance quality (35, 36). Third, digital government may enhance individuals’ sense of self-empowerment. E-participation channels, such as online political consultation and online complaint systems, can lower the threshold for residents to express demands and grievances, strengthening their sense of being responded to and their perceived efficacy in public participation (37, 38). These positive institutional experiences originating in the public sphere may contribute to higher levels of residents’ overall life satisfaction and life happiness.

Furthermore, life happiness can be transformed into an important psychological resource that helps resist mental health risks and promote psychological wellbeing. From the perspective of positive psychology and the two-continuum model of mental health, mental health does not merely refer to the absence of clinical symptoms such as depression and anxiety; it also involves the presence of positive emotions, psychological functioning, and social functioning (14). Higher levels of life happiness generally imply more positive life evaluations, more stable emotional regulation, and a stronger sense of environmental mastery. These psychological resources can enhance individuals’ resilience in coping with external stress and social uncertainty (39, 40). Conversely, lower life evaluations are often closely associated with mental health problems such as depression and anxiety and may predict subsequent moderate-to-severe depression and related adverse outcomes (41, 42).

Therefore, digital government may be associated with residents’ mental health through a pathway of “improved governance experience–positive life evaluation–better mental health.” Based on these arguments, this study proposes the following hypothesis:

*H3*: Life happiness may constitute a potential transmission mechanism between digital government development and residents’ mental health. Specifically, digital government development is expected to be associated with higher levels of residents’ life happiness, and higher life happiness is further expected to be associated with better mental health.

### Marginal improvement under macro-structural constraints: urban–rural heterogeneity and compensatory empowerment

The relationship between digital government and residents’ mental health is unlikely to be evenly distributed across urban and rural populations. Under China’s urban–rural dual structure, rural residents have long faced structural constraints such as insufficient public service provision, weaker accessibility to health resources, and limited channels for information acquisition (43, 44). Compared with urban residents, rural residents often bear higher spatial, temporal, and informational costs when accessing government services, medical care, social security, and other public services. Therefore, the role of digital government in rural areas may not be limited to the online extension of existing service channels; rather, it may also function as a supplement to, and partial compensation for, traditional shortcomings in public service provision (45).

From the perspective of the capability approach, the accessibility of public services concerns not only the supply of resources itself but also whether individuals can convert external resources into real capabilities for improving their living conditions and pursuing lives they have reason to value (15, 46). Through telemedicine, public health information dissemination, online social security services, and government service platforms, digital government may help overcome geographical distance and information barriers, improve rural residents’ access to health services and basic public services, and thereby alleviate life stress and health anxiety arising from resource shortages and service inconvenience (47, 48). Meanwhile, government information disclosure, online grievance feedback, and grassroots digital governance platforms may also help reduce information asymmetry and insufficient responsiveness in rural public affairs, strengthening rural residents’ sense of institutional responsiveness, social support, and control over daily life (49, 50).

Of course, the compensatory effect of digital government is not unconditional. The digital divide, insufficient digital literacy, and unequal access capacity may constrain some rural residents’ ability to convert digital governance resources into actual benefits (16, 51). Nevertheless, from the perspective of baseline differences in urban and rural public service provision, rural residents originally face more pronounced service gaps and institutional frictions. Once digital government effectively improves its access to public services and institutional responsiveness, the potential marginal room for improvement may be greater. Accordingly, the positive association between digital government development and residents’ mental health may be more stable or more evident among rural residents. Based on these arguments, this study proposes the following hypothesis:

*H4*: The positive association between digital government development and residents’ mental health may vary between urban and rural residents and is expected to be more stable among rural residents.

**Figure 1:**
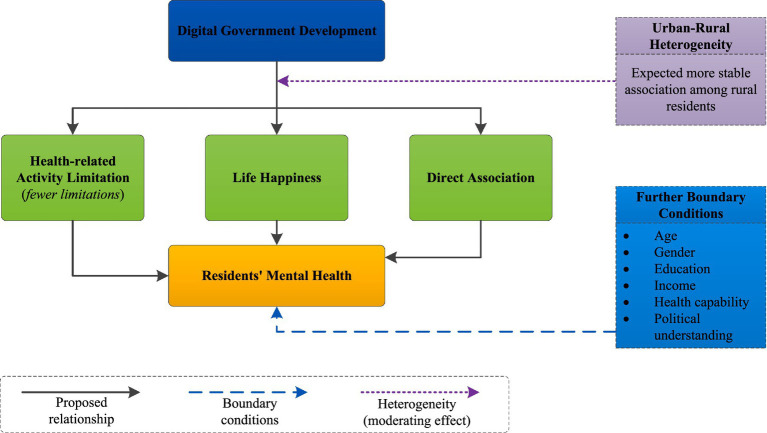
Conceptual framework of digital government and residents’ mental health. The figure summarizes the conceptual framework of this study. Digital government development is expected to be positively associated with residents’ mental health through two potential transmission pathways—health-related activity limitation and life happiness—as well as a direct association. Urban–rural heterogeneity and further boundary conditions may shape the strength and stability of this association.

The overall conceptual framework integrating the main association, the two potential transmission mechanisms, and urban–rural heterogeneity is presented in Figure 1.

## Methodology

### Data and sample construction

This study adopts a pooled cross-sectional research design that links provincial-level digital government development indicators with individual-level survey data to examine the relationship between digital government and residents’ mental health. The individual-level data are drawn from the 2021 and 2023 waves of the Chinese General Social Survey (CGSS), while the macro-level data are obtained from the *China Digital Government Development Index Report* published by the Data Governance Research Center, School of Social Sciences, Tsinghua University. The core explanatory variable is the provincial Digital Government Development Index, and this study uses its standardized value to measure the level of digital government development across provinces.

To strengthen temporal ordering between variables and mitigate potential reverse causality concerns, this study adopts a one-period lagged matching strategy. Specifically, the 2020 provincial Digital Government Development Index is matched with individual respondents from the 2021 CGSS, and the 2022 provincial Digital Government Development Index is matched with individual respondents from the 2023 CGSS. The matching is conducted according to respondents’ province of residence, thereby constructing a pooled cross-sectional dataset that combines provincial-level digital government indicators with individual-level survey information.

The merged raw sample contains 19,474 individual observations, including 8,148 observations from the 2021 CGSS and 11,326 observations from the 2023 CGSS. Except for a small number of cases that could not be matched with the digital government index because provincial information could not be identified, the vast majority of respondents were successfully matched at the provincial level. After further excluding observations with missing values in mental health, the digital government index, and major control variables, the effective analytical sample for the baseline regression consists of 17,585 observations covering 31 provincial-level administrative units. Among them, 7,162 observations are from the 2021 wave, and 10,423 observations are from the 2023 wave. In addition, because different empirical models involve different sets of variables, the effective sample size varies slightly across analyses due to variable-specific missingness. The actual number of observations used in each model is therefore reported in the corresponding regression tables.

### Measures

Dependent variable. The dependent variable in this study is residents’ mental health. It is measured using the CGSS item: “In the past four weeks, how often have you felt depressed or dejected?” The original responses are coded on a five-point scale and recoded in a positive direction for the empirical analysis. Higher values indicate better mental health, that is, a lower frequency of feeling depressed or dejected.

Core explanatory variable. The core explanatory variable is the provincial Digital Government Development Index. This indicator is drawn from the *China Digital Government Development Index Report* and is used to capture the level of digital government development across provinces. The index comprehensively reflects provincial digital government development across multiple dimensions, including organizational structure, institutional systems, governance capacity, and governance outcomes. To facilitate coefficient interpretation and comparability across variables, the Digital Government Development Index is standardized, and its standardized value is used in the regression models.

Potential transmission variables. To examine the potential transmission mechanisms between digital government and residents’ mental health, this study includes two mediating variables: health-related activity limitation and life happiness. First, health-related activity limitation reflects the extent to which residents are restricted in work, daily life, or social participation due to physical health problems. Following the coding direction of the variable, this study treats it as a positive health indicator: higher values indicate better physical health and fewer health-related activity limitations. This variable is used to examine whether digital government may be linked to better mental health by improving the accessibility of public and health services and thereby alleviating residents’ health-related activity limitations. Second, life happiness captures residents’ overall subjective evaluation of their living conditions. Higher values indicate higher levels of life happiness. This variable is used to examine whether digital government may be associated with residents’ mental health by improving public service experiences, strengthening perceived governance responsiveness, and enhancing overall life evaluation.

Control variables. To reduce potential confounding from individual characteristics and regional development differences, this study includes a set of individual-level and macro-level control variables. Individual-level controls include gender, age, age squared, urban–rural type, years of education, the logarithm of annual personal income, household car ownership, medical insurance participation, whether BMI falls within the normal range, and marital status. At the macro level, this study controls for the standardized value of provincial GDP per capita to account for regional differences in economic development.

## Empirical strategy

### Baseline model specification

Given the pooled cross-sectional structure of the data, this study uses ordinary least squares (OLS) regression as the baseline estimation strategy to examine the relationship between digital government development and residents’ mental health, controlling for region fixed effects and year fixed effects. The model is specified as follows:


$$\text{MentalHealt}h_{\mathrm{ijt}}=\beta_{1}\text{DigitalGo}v_{\mathrm{jt}}+\beta_{2}X_{\mathrm{ijt}}+\mu_{j}+\lambda_{t}+\varepsilon_{\mathrm{ijt}}$$

Where $\text{MentalHealt}h_{\mathrm{ijt}}$ denotes the mental health status of individual i in region j in year t ; $\text{DigitalGo}v_{\mathrm{jt}}$ denotes the level of digital government development in region j ; and $X_{\mathrm{ijt}}$ is a vector of individual- and region-level control variables, including gender, age and age squared, urban–rural type, years of education, the logarithm of personal income, household car ownership, medical insurance participation, normal BMI status, marital status, and the standardized value of provincial GDP per capita. $\mu_{j}$ represents region fixed effects, which absorb time-invariant regional endowments, while $\lambda_{t}$ represents year fixed effects, which control for common macro-level time shocks. $\varepsilon_{\mathrm{ijt}}$ is the random error term.

Although the dependent variable, mental health, is ordinal in nature, OLS is used as the baseline estimator because of its computational advantages in models with high-dimensional fixed effects and the intuitive interpretation of its marginal coefficients. Because the core explanatory variable is measured at the provincial level, the main regressions use province-clustered robust standard errors, unless otherwise specified, to address potential within-province correlation in the error terms.

To further examine whether the association between digital government and mental health varies under China’s urban–rural dual structure, this study not only estimates subgroup regressions but also introduces an interaction term between the Digital Government Development Index and urban–rural type in the full sample. The model is specified as follows:


$$\begin{array}{l} \text{MentalHealt}h_{\mathrm{ijt}}=\gamma_{1}\text{DigitalGo}v_{\mathrm{jt}}+\gamma_{2}\text{UrbanRura}l_{\mathrm{ijt}}\\ +\gamma_{3}(\text{DigitalGo}v_{\mathrm{jt}}\times \text{UrbanRura}l_{\mathrm{ijt}})+\gamma_{4}X_{\mathrm{ijt}}^{\prime }+\mu_{j}+\lambda_{t}+\varepsilon_{\mathrm{ijt}} \end{array}$$

Where $\text{UrbanRura}l_{\mathrm{ijt}}$ is a dummy variable indicating urban–rural type. The coefficient of the interaction term, $\gamma_{3}$ , is used to test whether the relationship between digital government development and residents’ mental health differs by urban–rural status, thereby providing an empirical test of the compensatory empowerment hypothesis. $X_{\mathrm{ijt}}^{\prime }$ denotes the vector of control variables excluding urban–rural type.

### Endogeneity discussion and instrumental variable (2SLS) estimation

Although the baseline model controls for two-way fixed effects and a rich set of covariates, the relationship between digital government development and residents’ mental health may still be subject to endogeneity concerns arising from omitted variables, measurement error, or reverse causality. To address these concerns, this study employs a two-stage least squares (2SLS) strategy and constructs two instrumental variables.

The first instrumental variable is the spatial lag of digital government development in neighboring provinces (IV1). Local governments’ digital transformation is strongly shaped by interregional yardstick competition and policy diffusion. Therefore, the level of digital government development in neighboring provinces is expected to be highly correlated with local digital government development, satisfying the relevance condition. At the same time, after controlling for individual characteristics and local macroeconomic conditions, the level of government digitalization in neighboring provinces is unlikely to directly affect the individual-level mental health of local residents, which supports the plausibility of the exogeneity condition.

The second instrumental variable is a historical shift-share, or Bartik, instrument (IV2). Specifically, this study uses the number of post offices in each province in 1982 as the historical share and interacts it with the lagged national number of internet users as the shift component. This instrument captures the path dependence of contemporary digital development on historical communication infrastructure. Because the number of post offices in 1982 is temporally distant from the observation period of this study, its association with contemporary individual mental health is more likely to operate through subsequent communication infrastructure and digital development trajectories rather than through a direct channel.

The two-stage models are specified as follows:

First stage:


$$\text{DigitalGo}v_{\mathrm{jt}}=\pi_{1}\mathrm{IV}1_{\mathrm{jt}}+\pi_{2}\mathrm{IV}2_{\mathrm{jt}}+\pi_{3}X_{\mathrm{ijt}}+\lambda_{t}+\nu_{\mathrm{ijt}}$$

Second stage:


$$\text{MentalHealt}h_{\mathrm{ijt}}=\alpha_{1}{\hat{\text{DigitalGov}}}_{\mathrm{jt}}+\alpha_{2}X_{\mathrm{ijt}}+\lambda_{t}+\eta_{\mathrm{ijt}}$$

Where ${\hat{\text{DigitalGov}}}_{\mathrm{jt}}$ denotes the fitted value obtained from the first-stage estimation. The Kleibergen–Paap rk *F* statistic is reported to assess potential weak-instrument concerns, and the Hansen *J* statistic is used to test the overidentifying restrictions. It should be acknowledged that historical communication infrastructure may still indirectly affect contemporary health through long-term economic evolution or social capital accumulation, which makes the exclusion restriction difficult to establish perfectly. Therefore, this study treats the 2SLS results as important supplementary evidence for the baseline findings rather than as the sole basis for causal inference.

### Robustness checks

To ensure the reliability of the findings, this study conducts four sets of robustness checks.

First, the dependent variable is replaced. Specifically, mental health is replaced with self-rated health to examine whether digital government development is also consistently associated with residents’ general health evaluations. Self-rated health reflects residents’ subjective assessment of their overall health status, with higher values indicating better self-rated health.

Second, an alternative nonlinear estimation model is used. Given the ordinal nature of the dependent variable, this study re-estimates the baseline specification using an Ordered Probit model to verify that the main findings are not driven by the linearity assumption of OLS.

Third, a hierarchical linear model (HLM) is estimated. Because individual respondents are nested within macro-level regions, this study estimates a multilevel model with regional random intercepts to better account for unobserved heterogeneity arising from the cross-level data structure.

Fourth, observations from municipalities directly under the central government are excluded. Considering that Beijing, Shanghai, Tianjin, and Chongqing differ from other regions in administrative status, digital infrastructure, and resource allocation, the models are re-estimated after excluding these observations to ensure that the results are not driven by these administratively distinctive cases.

### Mechanism exploration and multidimensional heterogeneity analysis

To explore the potential mechanisms linking digital government and residents’ mental health, this study draws on the channel analysis approach commonly used in applied microeconometrics. Specifically, it focuses on two transmission paths: health-related activity limitation as a physiological pathway, and life happiness as a psychological resource pathway.

First, this study examines whether digital government development is significantly associated with the proposed mechanism variables, that is, the ( X→Mediator ) relationship:


$$\text{Mediato}r_{\mathrm{ijt}}=\theta_{1}\text{DigitalGo}v_{\mathrm{jt}}+\theta_{2}X_{\mathrm{ijt}}+\mu_{j}+\lambda_{t}+\varepsilon_{\mathrm{ijt}}$$

Second, the core explanatory variable and the mechanism variable are jointly included in the baseline model:


$$\begin{array}{l} \text{MentalHealt}h_{\mathrm{ijt}}=\delta_{1}\text{DigitalGo}v_{\mathrm{jt}}+\delta_{2}\text{Mediato}r_{\mathrm{ijt}}\\ +\delta_{3}X_{\mathrm{ijt}}+\mu_{j}+\lambda_{t}+\varepsilon_{\mathrm{ijt}} \end{array}$$

If digital government development is significantly associated with the mechanism variable, the mechanism variable is significantly associated with mental health after being included in the model, and the coefficient of digital government development decreases relative to the baseline model, then the results would be consistent with the variable serving as a potential mechanism pathway. It should be emphasized that, given the inherent limitations of cross-sectional data, this mechanism analysis is intended to provide empirical support for the proposed logical chain rather than to identify a causal mediation effect in a strict sense.

For heterogeneity analysis, beyond the core examination of urban–rural differences, this study further divides both the full sample and the rural resident sample across multiple dimensions, including demographic characteristics (age and gender), socioeconomic status (educational attainment, income level, and region), physiological health endowment (degree of health-related activity limitation), and cognitive capital, proxied by political understanding. By comparing the direction, statistical significance, and confidence intervals of the coefficients of digital government development across different subsamples, this analysis aims to further identify the groups for whom the positive association between digital government development and mental health is more evident, as well as the boundary conditions of this association.

## Results

### Descriptive statistics and urban–rural comparisons

Table 1 reports the descriptive statistics for the main variables. Continuous and ordinal variables are reported with the number of observations, mean, standard deviation, minimum, and maximum values, while categorical variables are reported with frequencies and percentages. The variables cover residents’ mental health, provincial digital government development, health-related activity limitation, self-rated health, life happiness, and the main control variables.

**Table 1:** Descriptive statistics of main variables.

Variable	Observations	Mean	SD	Min	Max
Panel A: Continuous and ordinal variables					
Mental health	17,585	3.773	1.084	1	5
Digital government index (standardized)	17,585	0.021	0.992	−2.056	1.354
Digital government index (raw value)	17,585	65.034	7.285	49.773	74.822
Health-related activity limitation	17,557	3.779	1.183	1	5
Self-rated health	17,578	3.422	1.062	1	5
Life happiness	15,186	3.913	0.825	1	5
Age	17,535	49.767	16.986	18	99
Years of education	17,585	10.546	4.788	0	19
Log personal income	17,585	8.668	4.061	0	16.117
GDP per capita (standardized)	17,585	−0.002	0.996	−1.780	2.052
Spatial-lag instrumental variable	17,585	64.888	6.667	51.365	74.185
Historical shift-share instrumental variable	17,585	0.810	1.597	0.008	8.354

Variable	Category	Frequency	Percentage
Panel B: Categorical variables			
Survey year	2021	7,162	40.73%
	2023	10,423	59.27%
Gender	Male	8,977	51.05%
	Female	8,608	48.95%
Household car ownership	No	8,666	49.28%
	Yes	8,919	50.72%
Medical insurance	No	1,004	5.71%
	Yes	16,581	94.29%
BMI status	Abnormal	8,463	48.13%
	Normal	9,122	51.87%
Urban–rural type	Urban residents	8,127	46.22%
	Rural residents	9,458	53.78%
Marital status	Unmarried	5,263	29.93%
	Married	12,322	70.07%

Mental health, health-related activity limitation, self-rated health, and life happiness are coded in a positive direction. Higher values indicate better mental health, fewer health-related activity limitations, better self-rated health, and higher life happiness, respectively. The digital government index and GDP per capita are standardized variables. The historical shift-share instrumental variable is scaled in units of 100 million. Percentages for categorical variables are calculated based on the baseline regression sample.

The baseline regression sample includes 17,585 observations, of which 7,162 are from the 2021 wave, and 10,423 are from the 2023 wave. The mean value of residents’ mental health is 3.773, with a standard deviation of 1.084. Because this variable is coded in a positive direction, higher values indicate a lower frequency of feeling depressed or dejected. This suggests that the overall mental health level of the sample is above the midpoint. The core explanatory variable is the provincial Digital Government Development Index, whose standardized value is used in the subsequent regression analyses. The standardized digital government index has a mean of 0.021 and a standard deviation of 0.992, indicating a relatively stable distribution after standardization.

Regarding the mechanism and health-related variables, the mean value of health-related activity limitation is 3.779. It should be noted that this variable is also coded in a positive direction, meaning that higher values indicate fewer restrictions in work and daily activities caused by health problems. The mean value of self-rated health is 3.422, and the mean value of life happiness is 3.913, suggesting that respondents report moderately high levels of general health evaluation and life happiness. With respect to the control variables, the average age of respondents is 49.767 years, the average years of education is 10.546, and the mean logarithm of personal income is 8.668. Among the binary variables, women account for 48.95% of the sample, respondents with household car ownership account for 50.72%, those participating in basic medical insurance account for 94.29%, respondents with normal BMI account for 51.87%, rural residents account for 53.78%, and married respondents account for 70.07%.

Furthermore, this study compares digital government exposure, mental health, and health-related outcomes across urban and rural groups, as reported in Appendix Table A1. The mean standardized digital government index matched to urban residents is 0.247, which is higher than that matched to rural residents (−0.173), and the difference is statistically significant. This indicates that, on average, urban residents are matched with provinces with higher levels of digital government development than rural residents.

Urban and rural residents also differ in mental health, health-related activity limitation, self-rated health, and life happiness. The mean mental health score of urban residents is 3.842, higher than that of rural residents, which is 3.713. The proportion of urban residents who always or often feel depressed is 10.0%, lower than the corresponding proportion among rural residents, which is 14.5%. In addition, the mean value of health-related activity limitation is 3.873 for urban residents and 3.698 for rural residents, indicating that urban residents experience relatively fewer health-related activity limitations. Urban residents also report higher levels of self-rated health and life happiness. Appendix Table A1 further shows that these urban–rural differences are statistically significant.

Overall, Table 1 and Appendix Table A1 indicate that the sample exhibits notable urban–rural differences in digital government exposure, mental health, health-related activity limitation, self-rated health, and life happiness. These descriptive patterns provide an important empirical background for the subsequent analysis of the relationship between digital government development and residents’ mental health, the potential mechanisms underlying this relationship, and heterogeneity by urban–rural type.

### Baseline regression results

Table 2 reports the baseline regression results. Column (1) presents the full-sample estimates without controlling for provincial GDP per capita, while Column (2) further includes provincial GDP per capita and serves as the preferred baseline specification. The results show that the coefficients of the standardized digital government index are positive and statistically significant in both models. In Column (2), after controlling for individual characteristics, regional economic development, year fixed effects, and region fixed effects, a one-standard-deviation increase in the digital government index is associated with an average increase of 0.268 points in residents’ mental health scores. This finding indicates a significant positive association between digital government development and residents’ mental health, providing preliminary empirical support for H1.

**Table 2:** Baseline regression results: digital government and mental health.

Variables/statistics	(1)	(2)
	Full sample without GDP	Full sample baseline
Digital government index	0.318^**^	0.268^**^
	(0.145)	(0.130)
Rural resident	−0.066^**^	−0.070^**^
	(0.032)	(0.030)
Female	−0.077^***^	−0.076^***^
	(0.019)	(0.019)
Age	−0.007^*^	−0.007^*^
	(0.004)	(0.004)
Age squared	0.000^**^	0.000^**^
	(0.000)	(0.000)
Years of education	0.011^***^	0.011^***^
	(0.003)	(0.002)
Log personal income	0.016^***^	0.017^***^
	(0.003)	(0.003)
Household car ownership	0.103^***^	0.103^***^
	(0.022)	(0.022)
Medical insurance	0.010	0.010
	(0.032)	(0.033)
Normal BMI	0.036^**^	0.036^**^
	(0.016)	(0.016)
Married	0.127^***^	0.127^***^
	(0.022)	(0.021)
GDP per capita		−0.027
		(0.042)
Constant	3.807^***^	3.807^***^
	(0.102)	(0.100)
Year fixed effects	Yes	Yes
Region fixed effects	Yes	Yes
Observations	17,535	17,535
Adjusted *R*^2^	0.039	0.040

Standard errors clustered at the provincial level are reported in parentheses. The dependent variable is mental health, coded so that higher values indicate better mental health. The digital government index is standardized. “Rural resident” equals 1 for rural residents and 0 for urban residents. Column (1) excludes provincial GDP per capita; Column (2) includes GDP per capita and serves as the preferred baseline specification. ^*^p < 0.10, ^**^p < 0.05, ^***^p < 0.01.

Regarding the control variables in the full-sample models, years of education, personal income, household car ownership, normal BMI status, and being married are generally positively associated with better mental health. The coefficient of rural resident status is negative and statistically significant, suggesting that, after controlling for other covariates, rural residents report lower mental health scores than urban residents.

### Heterogeneity by urban–rural type

Table 3 reports the heterogeneity analysis by urban–rural type. Columns (1) and (2) present subgroup estimates for rural and urban residents, respectively, while Column (3) reports the interaction model based on the full sample. The subgroup results show that the coefficient of the digital government index is positive and statistically significant among rural residents (*β* = 0.383, *p* < 0.01), whereas the coefficient is smaller and statistically insignificant among urban residents (*β* = 0.027). These results suggest that the positive association between digital government development and residents’ mental health is mainly observed in the rural resident sample, which is broadly consistent with the theoretical expectation of H4 that this association may be more stable or more evident among rural residents.

**Table 3:** Urban–rural heterogeneity analysis: digital government and mental health.

Variables/statistics	(1)	(2)	(3)
	Rural residents	Urban residents	Interaction model
Digital government index	0.383^***^	0.027	0.217
	(0.138)	(0.139)	(0.129)
Rural resident			−0.071^**^
			(0.029)
Digital government index × Rural resident			0.049
			(0.033)
Total effect for rural residents			0.267^*^
			(0.131)
*p*-value for total effect			0.050
Female	−0.111^***^	−0.056^**^	−0.075^***^
	(0.021)	(0.026)	(0.019)
Age	−0.005	0.000	−0.006
	(0.005)	(0.005)	(0.004)
Age squared	0.000	0.000	0.000^**^
	(0.000)	(0.000)	(0.000)
Years of education	0.010^**^	0.010^***^	0.011^***^
	(0.004)	(0.004)	(0.002)
Log personal income	0.016^***^	0.008^*^	0.016^***^
	(0.003)	(0.004)	(0.003)
Household car ownership	0.166^***^	0.047	0.102^***^
	(0.023)	(0.032)	(0.022)
Medical insurance	0.005	0.028	0.011
	(0.039)	(0.048)	(0.033)
Normal BMI	0.052^**^	0.021	0.036^**^
	(0.023)	(0.020)	(0.016)
Married	0.142^***^	0.096^***^	0.127^***^
	(0.028)	(0.031)	(0.021)
GDP per capita	−0.040	−0.032	−0.024
	(0.040)	(0.050)	(0.042)
Constant	3.825^***^	3.623^***^	3.792^***^
	(0.153)	(0.108)	(0.095)
Year fixed effects	Yes	Yes	Yes
Region fixed effects	Yes	Yes	Yes
Observations	9,437	8,098	17,535
Adjusted *R*^2^	0.042	0.047	0.040

Province-clustered standard errors are reported in parentheses. The dependent variable is mental health, coded so that higher values indicate better mental health. The digital government index is standardized. Columns (1) and (2) report rural and urban subgroup estimates, respectively; Column (3) reports the interaction model. “Rural resident” equals 1 for rural residents and 0 for urban residents. The total effect for rural residents is calculated using a linear combination test. ^*^p < 0.10, ^**^p < 0.05, ^***^p < 0.01.

To further examine whether the observed urban–rural difference is statistically meaningful, Column (3) introduces an interaction term between the digital government index and rural resident status. The coefficient of the interaction term is positive but statistically insignificant (*β* = 0.049), indicating that the coefficient difference between rural and urban residents does not reach conventional levels of statistical significance. However, the linear combination test shows that the total effect of the digital government index for rural residents remains positive and marginally significant (*β* = 0.267, *p* = 0.050).

Therefore, the interpretation of H4 should remain cautious. While the subgroup results are consistent with the direction of the compensatory empowerment hypothesis and suggest that the positive association between digital government development and mental health is more stable among rural residents, the interaction model does not provide strong statistical evidence that the urban–rural difference is significant. Accordingly, H4 receives directional or partial empirical support rather than full confirmation.

### Robustness checks

To further assess the reliability of the baseline findings, this study conducts robustness checks from four perspectives: using an alternative outcome variable, adopting an alternative model specification, accounting for the nested cross-level data structure, and excluding administratively distinctive cases. The results are reported in Table 4.

**Table 4:** Robustness checks.

Variables/statistics	Full sample	Rural residents	Urban residents
Panel A. Alternative dependent variable: self-rated health			
Digital government index	0.182^**^	0.287^***^	−0.021
	(0.085)	(0.083)	(0.124)
Observations	17,583	9,470	8,113
Adjusted *R*^2^	0.110	0.143	0.071
Panel B. Ordered Probit model			
Digital government index	0.321^**^	0.430^***^	0.063
	(0.138)	(0.144)	(0.154)
Observations	17,535	9,437	8,098
Pseudo *R*^2^	0.015	0.016	0.020
Panel C. Hierarchical linear model			
Digital government index	0.238^***^	0.231^***^	0.135^**^
	(0.048)	(0.054)	(0.056)
Observations	17,535	9,437	8,098
Wald *χ*^2^	719.125	414.197	404.030
Panel D. Excluding municipalities			
Digital government index	0.303^**^	0.365^**^	0.122
	(0.125)	(0.144)	(0.135)
Observations	14,338	8,543	5,795
Adjusted *R*^2^	0.042	0.040	0.053
Individual controls	Yes	Yes	Yes
Year fixed effects	Yes	Yes	Yes
Region fixed effects	Yes	Yes	Yes

Entries report the estimated coefficients of the standardized digital government index, with standard errors in parentheses. Panel A uses self-rated health as the dependent variable. Panel B reports Ordered Probit estimates. Panel C reports hierarchical linear models with province-level random intercepts. Panel D excludes observations from Beijing, Shanghai, Tianjin, and Chongqing. Panels A, B, and D include individual controls, year fixed effects, and region fixed effects; Panel C includes individual controls, year fixed effects, and province-level random intercepts. Standard errors are clustered at the provincial level where applicable. ^*^p < 0.10, ^**^p < 0.05, ^***^p < 0.01.

First, Panel A uses an alternative core outcome variable. Given the close relationship between mental health and individuals’ overall health evaluation, the dependent variable is replaced with self-rated health. The results show that the coefficient of the digital government index remains positive and statistically significant in the full sample and the rural resident sample, while it is statistically insignificant in the urban resident sample. This suggests that the positive association between digital government development and residents’ health-related outcomes is not limited to the mental health dimension and is more stable among rural residents.

Second, Panel B adopts an alternative nonlinear estimation model. Considering that the dependent variable, mental health, is measured as a five-category ordinal variable, this study re-estimates the model using an ordered probit model. The results show that the coefficient of the digital government index remains positive and statistically significant in the full sample and the rural resident sample, but not in the urban resident sample. This pattern is broadly consistent with the baseline regression results, indicating that the main findings do not depend on the linear specification of OLS.

Third, Panel C reports the results from a hierarchical linear model (HLM). Because individual respondents are nested within provinces, this study further estimates a multilevel linear model with province-level random intercepts. The results show that the digital government index is positively and significantly associated with mental health in the full sample, the rural resident sample, and the urban resident sample. This indicates that the positive relationship between digital government development and residents’ mental health remains after accounting for province-level random heterogeneity. It should be noted that, although the coefficient for the urban resident sample also reaches statistical significance in this specification, the baseline regression, alternative outcome test, and ordered probit results collectively suggest that the positive association between digital government and mental health is still more stable among rural residents.

Fourth, Panel D excludes observations from administratively distinctive municipalities. Considering that Beijing, Shanghai, Tianjin, and Chongqing differ from other regions in administrative status, resource allocation, public service provision, and the foundations of digital government development, the models are re-estimated after excluding these observations. The results show that the coefficient of the digital government index remains positive and statistically significant in the full sample and the rural resident sample, while it is statistically insignificant in the urban resident sample. This indicates that the baseline findings are not driven by a small number of municipalities directly under the central government.

Taken together, these robustness checks show that the positive association between digital government development and residents’ mental health is not sensitive to the use of an alternative outcome variable, a nonlinear model specification, adjustment for the cross-level data structure, or the exclusion of these specific municipalities. These results further support the basic finding that digital government development is positively associated with residents’ mental health and strengthen the robustness of the evidence for H1. At the same time, most robustness checks show that this positive association is more stable in the full sample and the rural resident sample, whereas the evidence for the urban resident sample is relatively less consistent across model specifications. This provides supplementary empirical evidence for H4, which anticipates that the positive association may be more stable among rural residents, but this interpretation should still be considered cautiously in light of the interaction results reported in Table 3.

### Instrumental variable estimates

Table 5 reports the two-stage least squares (2SLS) instrumental variable estimates. In the full sample, the coefficient of the digital government index remains positive and statistically significant (*β* = 0.166, *p* < 0.01), indicating that the positive association between digital government development and residents’ mental health persists after potential endogeneity concerns are addressed through the IV strategy. The estimated coefficients are also positive and statistically significant in both the rural and urban resident subsamples. The coefficient is larger for rural residents than for urban residents (0.181 vs. 0.123), a pattern that is broadly consistent with the compensatory empowerment perspective. However, because the difference between these two coefficients is not formally tested within this IV specification, this pattern should be interpreted as suggestive rather than conclusive evidence of urban–rural heterogeneity.

**Table 5:** Instrumental variable estimates: digital government and mental health.

Variables/statistics	(1)	(2)	(3)
	Full sample	Rural residents	Urban residents
Digital government index	0.166^***^	0.181^**^	0.123^**^
	(0.059)	(0.070)	(0.062)
Individual controls	Yes	Yes	Yes
GDP per capita	Yes	Yes	Yes
Year fixed effects	Yes	Yes	Yes
Observations	17,535	9,437	8,098
Kleibergen–Paap F	13.608	13.321	11.267
Hansen J *p*-value	0.212	0.289	0.174

Standard errors clustered at the provincial level are reported in parentheses. The dependent variable is mental health, coded so that higher values indicate better mental health. The digital government index is instrumented by the spatial lag of neighboring provinces’ digital government development and a historical shift-share instrument. Individual controls include gender, age, age squared, years of education, log personal income, household car ownership, medical insurance, normal BMI, marital status, and urban–rural type in the full-sample model. The 2SLS specifications include year fixed effects but do not include province fixed effects, given the limited within-province variation in the available two-wave structure. Full results with control variables are reported in Appendix Table A2. ^*^p < 0.10, ^**^p < 0.05, ^***^p < 0.01.

It is worth noting that the coefficient for urban residents, which is statistically insignificant in the baseline OLS estimation, becomes significant in the IV specification. One possible explanation is that unobserved factors may have attenuated the OLS estimate for the urban sample, resulting in a downward bias. Nevertheless, this result should be interpreted with caution, as the IV estimation is intended to provide supplementary evidence rather than to replace the baseline model specification.

The diagnostic statistics provide supportive evidence for the relevance of the instruments. The Kleibergen–Paap rk Wald *F*-statistics exceed the conventional rule-of-thumb threshold of 10 across all three models, suggesting that weak identification is not a major concern, although the statistic for the urban resident sample is relatively close to this threshold. Furthermore, the Hansen J test *p*-values range from 0.174 to 0.289, indicating that the overidentifying restrictions are not rejected. Overall, the 2SLS results provide additional evidence that the positive association between digital government development and residents’ mental health is unlikely to be entirely driven by reverse causality or omitted variable bias.

### Mechanism analysis

Table 6 reports the results for the two proposed transmission channels—health-related activity limitation and life happiness—which correspond to H2 and H3, respectively.

**Table 6:** Mechanism analysis: health-related activity limitation and life happiness.

Variables/statistics	(1)	(2)	(3)	(4)	(5)	(6)
	Total	Path A	Direct	Total	Path A	Direct
Digital government index	0.268^**^	0.345^***^	0.115	0.258^*^	0.240^***^	0.163
	(0.130)	(0.103)	(0.094)	(0.136)	(0.077)	(0.126)
Health-related activity limitation			0.442^***^			
			(0.010)			
Life happiness						0.393^***^
						(0.014)
Individual controls	Yes	Yes	Yes	Yes	Yes	Yes
Year fixed effects	Yes	Yes	Yes	Yes	Yes	Yes
Region fixed effects	Yes	Yes	Yes	Yes	Yes	Yes
Observations	17,507	17,507	17,507	15,136	15,136	15,136
Adjusted *R*^2^	0.040	0.091	0.251	0.032	0.059	0.116

Standard errors clustered at the provincial level are reported in parentheses. The dependent variable in Columns (1), (3), (4), and (6) is mental health. The dependent variable in Column (2) is health-related activity limitation, coded so that higher values indicate fewer health-related activity limitations. The dependent variable in Column (5) is life happiness, coded so that higher values indicate higher life happiness. “Total” reports the association between the digital government index and mental health. “Path A” reports the association between the digital government index and the mediator. “Direct” includes both the digital government index and the mediator in the mental health model. All models include the same set of individual controls as the baseline model, year fixed effects, and region fixed effects. ^*^p < 0.10, ^**^p < 0.05, ^***^p < 0.01.

Columns (1)–(3) examine the health-related activity limitation mechanism. Column (1) shows that, within the corresponding analytical sample, the digital government index is significantly and positively associated with residents’ mental health. Column (2) further shows that the digital government index is significantly associated with a higher score on the health-related activity limitation variable (*β* = 0.345, *p* < 0.01). Given the positive coding of this variable, where higher values indicate fewer activity limitations caused by health problems, this result suggests that digital government development is associated with a reduction in residents’ health-related activity limitations. Column (3) shows that, when both the digital government index and health-related activity limitation are included in the model, health-related activity limitation remains significantly and positively associated with mental health (*β* = 0.442, *p* < 0.01). Meanwhile, the coefficient of the digital government index decreases substantially from 0.268 in Column (1) to 0.115 and becomes statistically insignificant. These results are consistent with the argument that the alleviation of health-related activity limitations may serve as an important potential transmission pathway linking digital government development to residents’ mental health, thereby providing empirical support for H2.

Columns (4)–(6) examine the life happiness mechanism. Column (4) confirms the positive association between the digital government index and residents’ mental health within the corresponding analytical sample. Column (5) shows that the digital government index is significantly associated with higher levels of life happiness (*β* = 0.240, *p* < 0.01). Column (6) further indicates that life happiness is significantly and positively associated with mental health (*β* = 0.393, *p* < 0.01). At the same time, the coefficient of the digital government index decreases from 0.258 in Column (4) to 0.163 and becomes statistically insignificant. These results suggest that life happiness may also constitute a potential transmission pathway between digital government development and residents’ mental health, providing empirical support for H3.

Taken together, the results in Table 6 show that digital government development is not only positively associated with residents’ mental health but also associated with fewer health-related activity limitations and higher life happiness. After the two mechanism variables are incorporated into the mental health model, both are significantly and positively associated with mental health, while the coefficient of the digital government index declines and loses statistical significance. These findings provide stepwise regression evidence for H2 and H3. It should be emphasized that the mechanism analysis in this study is intended to examine whether the empirical relationships among digital government development, the proposed mechanism variables, and residents’ mental health are consistent with the theoretical expectations. Given the limitations of the pooled cross-sectional data structure, these results should be interpreted as empirical support for the proposed transmission pathways rather than as strict identification of causal mediation effects.

### Further heterogeneity analysis

**Figure 2:**
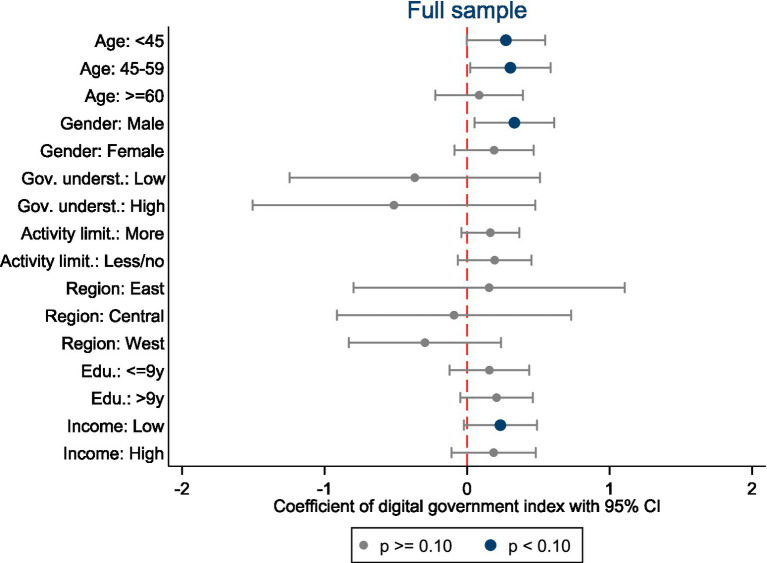
Further heterogeneity analysis: full sample.

**Figure 3:**
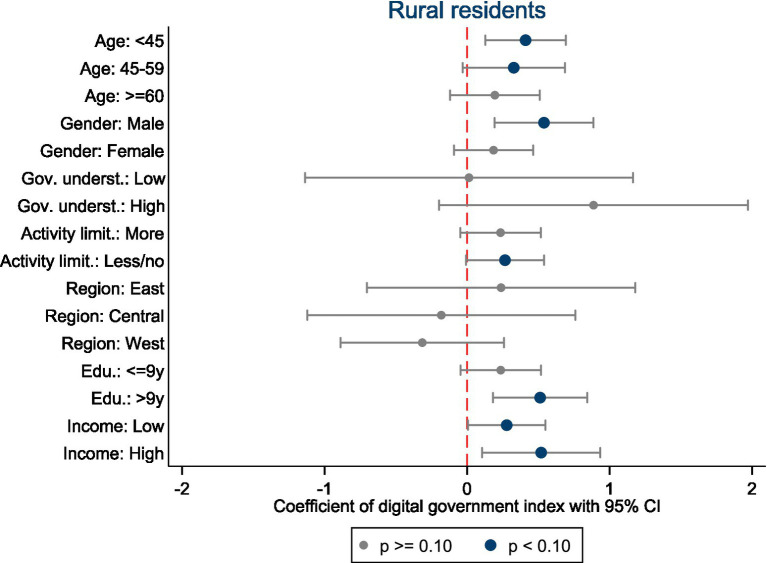
Further heterogeneity analysis: rural residents. The figures plot the estimated coefficients of the standardized digital government index and the corresponding 95% confidence intervals across subgroups. The dependent variable is mental health, coded so that higher values indicate better mental health. Blue markers indicate estimates significant at the 10% level, while gray markers indicate estimates not significant at the 10% level. The vertical dashed line represents zero. All models include the same set of controls as the baseline specification, year fixed effects, and region fixed effects. Standard errors are clustered at the provincial level.

To further identify the boundary conditions of the association between digital government and residents’ mental health, this study conducts a multidimensional heterogeneity analysis across demographic characteristics, socioeconomic status, physiological health constraints, regional location, and political understanding. Figures 2 and 3 plot the estimated coefficients of the digital government index and the corresponding 95% confidence intervals for the full sample and the rural resident sample, respectively. Overall, the positive association between digital government and mental health is not evenly distributed across all groups; rather, it appears to be shaped by individual resource endowments, health conditions, and the capacity to convert digital public services into meaningful benefits.

First, heterogeneity by demographic characteristics is relatively evident. In both the full sample and the rural resident sample, the positive association between the digital government index and mental health is mainly observed among residents under the age of 60 and among male residents. By contrast, the estimated coefficients for residents aged 60 and above and for female residents do not reach conventional levels of statistical significance. This pattern suggests that the potential health-related gains associated with digital government may be conditioned by age- and gender-related differences in digital capabilities. Older residents and some rural women may have relatively limited experience with digital devices, lower familiarity with online services, or weaker capacity to access and process public service information, which may make it more difficult for them to convert improvements in the digital governance environment into stable mental health-related gains. This finding is broadly consistent with digital divide research, which emphasizes disparities not only in access but also in effective use.

Second, the association between digital government and mental health may depend on thresholds in human capital and health capability. As shown in Figure 3, within the rural resident sample, the positive coefficient of the digital government index is mainly observed among residents with more than nine years of education and among those with fewer or no health-related activity limitations. By contrast, among rural residents with lower educational attainment or more severe health-related activity limitations, the confidence intervals of the estimates include zero. This suggests that the effective use of digital public services depends not only on the supply of digital platforms by the government but also on individuals’ comprehension, information-processing ability, and physical capacity. For rural residents with limited education or more severe activity limitations, even if digital government lowers institutional barriers to public service access, insufficient digital literacy, limited information-decoding capacity, or physical constraints may still prevent them from fully benefiting. Therefore, the compensatory role of digital government should not be understood as automatic; rather, it may be jointly constrained by human capital and health capability.

Third, the results by income level reveal a more complex pattern. In the full sample, the positive association between digital government and mental health is more evident among low-income groups. In the rural resident sample, however, both low-income and high-income groups show positive and statistically significant associations, with the coefficient slightly larger for the high-income group. This suggests that economic resources are not the only factor shaping the conversion of digital government development into mental health-related gains. For rural residents, once basic digital access and service-use capacity are in place, digital government may be associated with better mental health across different income groups. At the same time, the slightly larger coefficient among high-income rural residents suggests that economic advantage may further strengthen individuals’ ability to make use of digital public services, thereby amplifying the potential gains associated with digital governance.

Fourth, the estimates by regional location and political understanding are relatively unstable. The confidence intervals for these subgroups are generally wide, indicating limited precision after controlling for individual characteristics, regional economic development, year fixed effects, and region fixed effects. Therefore, strong conclusions should not be drawn from these subgroup estimates. Instead, they should be interpreted as supplementary clues for future research on the boundary conditions of the relationship between digital government and mental health.

Overall, the further heterogeneity analysis provides a more nuanced understanding of the boundary conditions of the compensatory empowerment perspective. The preceding results show that the positive association between digital government and mental health is more stable among rural residents. The present analysis further suggests that this association is not homogeneous within the rural population. Rather, it is more concentrated among groups with certain levels of human capital, health capability, and service-use capacity. In other words, digital government may create greater potential room for improvement among rural residents, but whether such potential can be translated into actual mental health-related gains still depends on individuals’ ability to access, understand, and use digital public services. Inclusive digital governance, therefore, should not stop at technological provision and platform coverage. It also needs to strengthen digital capability building, improve service comprehensibility, and provide offline auxiliary support for vulnerable groups, to avoid creating new usage barriers while improving public service accessibility.

## Discussion

Drawing on a matched dataset of the provincial Digital Government Development Index and micro-level data from the Chinese General Social Survey (CGSS), this study examines the relationship between digital government development and residents’ mental health, its potential transmission mechanisms, and urban–rural heterogeneity. Overall, the findings suggest three main conclusions. First, digital government development is positively associated with residents’ mental health. Second, the alleviation of health-related activity limitations and the enhancement of life happiness may serve as two potential transmission pathways. Third, this positive association appears more statistically stable within the rural resident sample, although evidence regarding the formal urban–rural difference should be interpreted cautiously.

First, this study finds a significant positive association between digital government development and residents’ mental health. This finding suggests that the social significance of digital government is not limited to improving administrative efficiency or optimizing service procedures; it may also be associated with better mental health among residents. Existing studies have mainly evaluated digital governance through e-government efficiency, public service satisfaction, and broad subjective wellbeing (4, 6), whereas this study extends the analysis to the more specific domain of mental health. Unlike general life satisfaction, which primarily captures individuals’ cognitive evaluation of overall quality of life, mental health involves positive emotional, psychological, and social functioning and is more directly related to experiences of psychological distress, such as depression and anxiety (33, 42, 52). The findings therefore suggest that improved institutional experiences generated by online services, data sharing, and process integration may not only enhance residents’ satisfaction with public services but also be linked to better mental health.

This finding can be understood through the lenses of administrative burden and institutional friction. In traditional public service settings, information asymmetry, fragmented procedures, and offline transaction costs may increase the time costs and cognitive burden associated with handling public affairs and may further generate frustration, helplessness, and uncertainty-related stress (9, 53). By providing online processing, data interoperability, process reengineering, and information disclosure, digital government may reduce these institutional frictions and provide residents with more accessible, transparent, and predictable service experiences. In this sense, the positive association between digital government and mental health reflects a potential link between improvements in the macro-level governance environment and micro-level psychological states.

Second, the mechanism analysis suggests that the alleviation of health-related activity limitations may serve as an important potential transmission pathway. The results show that digital government development is significantly associated with fewer health-related activity limitations, which are in turn positively associated with better mental health. Through online appointments, electronic health records, telemedicine, health information dissemination, and public health data integration, digital government may reduce the information, time, and transaction costs of accessing health services, thereby improving the accessibility and utilization of public health services (12, 25). Gallegos et al. (13) provide experimental evidence that digital appointment systems can increase the use of preventive health services by reducing access costs, while Jia (54) shows that governmental digital transformation promotes the utilization of basic public health services by improving health information accessibility.

Health-related activity limitation is important because it is not merely a matter of physical functioning. Physical limitations may undermine individuals’ capacity to fulfill social, family, and occupational roles, reduce self-esteem and sense of mastery, and limit opportunities for social participation, thereby contributing to depressive symptoms and psychological distress (55–57). Chronic illness and physical limitations may also disrupt the continuity of everyday life and become persistent stressors (27, 28). Therefore, if digital government improves the accessibility and continuity of health services and reduces activity limitations caused by health problems, it may also be associated with better mental health. This suggests that the psychological significance of digital government may come not only from administrative convenience but also from improved public health services, stronger continuity of care, and data-driven preventive health governance, which may help maintain physical functioning and reduce activity limitations (24–26). More broadly, recent studies on health development in China also show that socioeconomic conditions and public policy interventions can shape individual health through multiple channels, including medical care accessibility, working hours, social interaction, environmental quality, health awareness, and avoidance behavior (58, 59). These findings are consistent with the view that macro-level institutional and policy environments may be linked to individual health through broader changes in living conditions, behavioral constraints, and access to health-related resources.

Third, the enhancement of life happiness may constitute another potential transmission pathway. The mechanism results show that digital government development is significantly associated with higher life happiness, which is further associated with better mental health. By improving public service convenience, governance transparency, channels for grievance expression, and institutional responsiveness, digital government may improve residents’ overall evaluations of their living and institutional environments (37, 60). As a core component of subjective wellbeing, life happiness reflects individuals’ cognitive evaluation of their quality of life (33, 34). Higher life happiness generally indicates more positive life evaluations, a stronger sense of environmental mastery, and more stable emotional regulation, and is therefore associated with better mental health (14). Accordingly, the relationship between digital government and mental health may operate not only through objective health capability but also through positive life evaluation and the accumulation of psychological resources.

Finally, this study highlights the structural asymmetry of digital government’s mental health-related gains. Compared with urban residents, the positive association between digital government and mental health appears more statistically stable among rural residents. This empirical pattern provides directional and context-specific evidence for the compensatory empowerment hypothesis (15, 45, 46). Under China’s urban–rural dual structure, rural areas often face systemic constraints, including lagging public service provision, spatial mismatches in high-quality healthcare resources, and higher offline interaction costs shaped by geographical distance (50, 61, 65). Because the baseline public service foundation is relatively weaker in rural areas, digital government may function not merely as an additional service channel but as a compensatory response to structural service deficits. When online government services, telemedicine, and grassroots digital governance platforms help reduce spatiotemporal barriers and lower service access thresholds, they may create greater potential for improvement among rural residents. In terms of Sen’s capability approach, digital government may help expand rural residents’ capabilities to access resources and cope with risks, thereby making its positive association with mental health more stable in the rural sample.

However, the interpretation of urban–rural differences requires caution. The interaction model shows that the interaction term between the digital government index and rural resident status is positive but does not reach conventional levels of statistical significance. This means that although subgroup regressions and robustness checks indicate a more stable positive association in the rural sample, the current data do not provide strong statistical evidence of a significant difference between the urban and rural coefficients. Therefore, this study does not interpret H4 as fully confirmed; rather, it receives directional or partial empirical support. More precisely, this study finds that the positive association is more stable in the rural sample, but not that rural residents benefit significantly more than urban residents.

Further heterogeneity analysis also shows that the compensatory empowerment of digital government is not automatic. Even within the rural population, the positive association between digital government and mental health is more concentrated among residents with higher educational attainment, fewer or no health-related activity limitations, and a stronger capacity to understand and use services. This indicates that although digital government may lower supply-side institutional barriers, whether digital governance provision can be converted into actual health-related gains still depends on digital literacy, information-processing capacity, health capability, and service usability (62). Digital divide research also reminds us that digital access does not automatically translate into digital benefits. The health equity implications of digital government depend on whether platforms are accessible, usable, and understandable, and whether vulnerable groups receive necessary auxiliary support (16, 63, 64).

The findings of this study have both theoretical and practical implications. Theoretically, this study extends digital government research from administrative efficiency and service satisfaction to the field of mental health. By examining health-related activity limitation and life happiness as two potential transmission pathways, it shows that digital government may be linked to both residents’ objective health capability and subjective psychological resources. Practically, digital government development should not be evaluated solely by platform coverage or online processing rates. Greater attention should be paid to whether digital services actually improve the accessibility of public and health services, especially for rural residents. Future digital government initiatives should be more closely integrated with grassroots healthcare systems, improve age-friendly, barrier-free, and low-threshold platform design, and retain necessary offline fallback mechanisms to avoid creating new usage barriers while improving administrative efficiency.

Several limitations should be acknowledged. First, although this study uses lagged matching, robustness checks, and instrumental variable estimation to provide supplementary evidence, the pooled cross-sectional data structure limits strict causal inference. Second, the provincial Digital Government Development Index is a composite macro-level indicator and may not fully capture residents’ actual micro-level experiences with digital government services. Some components of the index may better reflect internal administrative capacity or institutional construction, whereas others may be more closely related to citizen-facing service usability, responsiveness, and accessibility. This may introduce measurement error when the index is used to represent residents’ actual exposure to digital government services. Such measurement error may also differ between urban and rural residents, because individuals living in the same province may experience substantially different levels of platform accessibility, service usability, and offline support. Therefore, the urban–rural heterogeneity results should be interpreted as evidence based on provincial-level digital government environments rather than as direct measures of individual digital service use. Third, the mechanism analysis is mainly based on stepwise regression and should therefore be interpreted as empirical support for potential transmission pathways rather than as a strict identification of causal mediation effects. Future research could combine individual-level data on digital government service use, evidence from specific public service scenarios, and longitudinal data to further examine the specific pathways linking digital government to residents’ mental health.

## Conclusion

This study links provincial-level digital government development with micro-level CGSS data to examine its association with residents’ mental health. The findings show that digital government development is positively associated with mental health, and that health-related activity limitation and life happiness may serve as two potential transmission pathways. The positive association appears more stable among rural residents, although the urban–rural difference should be interpreted cautiously. These results suggest that digital government may have implications beyond administrative efficiency, extending to public health and inclusive governance. Future digital government development should therefore pay greater attention to service accessibility, health service integration, digital inclusion, and offline support for vulnerable groups.

## Data Availability

Publicly available datasets were analyzed in this study. The micro-level data from the Chinese General Social Survey (CGSS) are available from the official project website at http://cgss.ruc.edu.cn/. The provincial-level Digital Government Development Index data were obtained from the publicly available China Digital Government Development Index Reports published by the Data Governance Research Center, School of Social Sciences, Tsinghua University.
