# Supplementary material for: Digital government and residents’ mental health in China: evidence on potential mechanisms and urban–rural heterogeneity
Source: Front Public Health. 2026 Jun 26;14:1872844. doi: 10.3389/fpubh.2026.1872844 (PMC13350043; doi:10.3389/fpubh.2026.1872844)
Supplement: Supplementary file 1 [file Table_1.docx]

**Appendix Table A1. Descriptive Statistics by Urban–Rural Type**

| **Variable** | **Urban residents** | **Rural residents** | **Difference** | **p-value** |
| --- | --- | --- | --- | --- |
| Digital government index (standardized) | 0.247 | -0.173 | 0.420 | <0.001 |
| Mental health | 3.842 | 3.713 | 0.129 | <0.001 |
| Always or often depressed (%) | 10.0% | 14.5% | -4.5 p.p. | <0.001 |
| Health-related activity limitation | 3.873 | 3.698 | 0.175 | <0.001 |
| Self-rated health | 3.471 | 3.379 | 0.092 | <0.001 |
| Life happiness | 3.972 | 3.861 | 0.111 | <0.001 |

***Notes:*** *Difference is calculated as the mean or percentage for urban residents minus that for rural residents. “Always or often depressed” reports the percentage-point difference, while all other rows report mean differences. The p-value for “Always or often depressed” is based on a two-sample test of proportions; p-values for the remaining variables are based on two-sample t-tests. Mental health, health-related activity limitation, self-rated health, and life happiness are positively coded, with higher values indicating better mental health, fewer health-related activity limitations, better self-rated health, and higher life happiness, respectively.*
