# Supplementary material for: Digital government and residents’ mental health in China: evidence on potential mechanisms and urban–rural heterogeneity
Source: Front Public Health. 2026 Jun 26;14:1872844. doi: 10.3389/fpubh.2026.1872844 (PMC13350043; doi:10.3389/fpubh.2026.1872844)
Supplement: Supplementary file 2 [file Table_2.docx]

**Appendix Table A2. Instrumental Variable Regression: Digital Government and Mental Health**

|  | (1) | (2) | (3) |
| --- | --- | --- | --- |
|  | Full sample | Rural residents | Urban residents |
| Digital government index | 0.166^***^ | 0.181^**^ | 0.123^**^ |
|  | (0.059) | (0.070) | (0.062) |
| Female | -0.078^***^ | -0.112^***^ | -0.058^**^ |
|  | (0.019) | (0.022) | (0.026) |
| Age | -0.007^*^ | -0.005 | 0.000 |
|  | (0.004) | (0.005) | (0.005) |
| Age squared | 0.000^**^ | 0.000 | 0.000 |
|  | (0.000) | (0.000) | (0.000) |
| Years of education | 0.011^***^ | 0.009^**^ | 0.010^***^ |
|  | (0.002) | (0.004) | (0.004) |
| Log personal income | 0.016^***^ | 0.016^***^ | 0.007^*^ |
|  | (0.003) | (0.003) | (0.004) |
| Household car ownership | 0.102^***^ | 0.165^***^ | 0.049 |
|  | (0.021) | (0.022) | (0.032) |
| Medical insurance | 0.016 | 0.012 | 0.031 |
|  | (0.031) | (0.037) | (0.046) |
| Normal BMI | 0.039^**^ | 0.058^***^ | 0.023 |
|  | (0.016) | (0.022) | (0.019) |
| Married | 0.130^***^ | 0.144^***^ | 0.099^***^ |
|  | (0.021) | (0.027) | (0.031) |
| GDP per capita | -0.070 | -0.096^**^ | -0.048 |
|  | (0.046) | (0.048) | (0.052) |
| Urban–rural type | -0.065^**^ |  |  |
|  | (0.031) |  |  |
| Constant | 3.854^***^ | 3.875^***^ | 3.689^***^ |
|  | (0.100) | (0.159) | (0.106) |
| Year fixed effects | Yes | Yes | Yes |
| Observations | 17535 | 9437 | 8098 |
| Kleibergen-Paap F | 13.608 | 13.321 | 11.267 |
| Hansen J p-value | 0.212 | 0.289 | 0.174 |

*Standard errors in parentheses*

** p < 0.1, ** p < 0.05, *** p < 0.01*
